# Predictive modelling of metabolic syndrome in Ghanaian diabetic patients: an ensemble machine learning approach

**DOI:** 10.1007/s40200-024-01491-7

**Published:** 2024-08-28

**Authors:** Emmanuel Acheampong, Eric Adua, Christian Obirikorang, Enoch Odame Anto, Emmanuel Peprah-Yamoah, Yaa Obirikorang, Evans Adu Asamoah, Victor Opoku-Yamoah, Michael Nyantakyi, John Taylor, Tonnies Abeku Buckman, Maryam Yakubu, Ebenezer Afrifa-Yamoah

**Affiliations:** 1 Leicester Cancer Research Centre, Department of Genetic and Genome Biology, University of Leicester, Leicester, UK; 2 Institute of Precision Health, University of Leicester, Leicester, UK; 3 Rural Clinical School, Medicine and Health, University of New South Wales, Sydney, NSW Australia; 4 School of Medical and Health Sciences, Edith Cowan University, 270 Joondalup Drive, Joondalup, WA 6027 Australia; 5 Department of Molecular Medicine, School of Medicine and Dentistry, Kwame Nkrumah University of Science and Technology, Kumasi, Ghana; 6 Department of Medical Diagnostics, Kwame Nkrumah University of Science and Technology, Kumasi, Ghana; 7 Teva Pharmaceuticals, Salt Lake City, UT USA; 8 Department of Nursing, Faculty of Health Sciences, Garden City University College (GCUC), Kenyasi, Kumasi, Ghana; 9 School of Optometry and Vision Science, University of Waterloo, Waterloo, Canada; 10 Department of Medical Laboratory Science, KAAF University College, Buduburam, Ghana; 11 Laboratory Department, Effia-Nkwanta Regional Hospital, Western Region, Takoradi, Ghana; 12 Mathematical Applications & Data Analytics Group, School of Science, Edith Cowan University, Perth, Australia

**Keywords:** Machine learning, Predictive modelling, Risk factors, Metabolic syndrome, Type 2 diabetes mellitus

## Abstract

**Objectives:**

The burgeoning prevalence of cardiometabolic disorders, including type 2 diabetes mellitus (T2DM) and metabolic syndrome (MetS) within Africa is concerning. Machine learning (ML) techniques offer a unique opportunity to leverage data-driven insights and construct predictive models for MetS risk, thereby enhancing the implementation of personalised prevention strategies. In this work, we employed ML techniques to develop predictive models for pre-MetS and MetS among diabetic patients.

**Methods:**

This multi-centre cross-sectional study comprised of 919 T2DM patients. Age, gender, novel anthropometric indices along with biochemical measures were analysed using BORUTA feature selection and an ensemble majority voting classification model, which included logistic regression, k-nearest neighbour, Gaussian Naive Bayes, Gradient boosting classification, and support vector machine.

**Results:**

Distinct metabolic profiles and phenotype clusters were associated with MetS progression. The BORUTA algorithm identified 10 and 16 significant features for pre-MetS and MetS prediction, respectively. For pre-MetS, the top-ranked features were lipid accumulation product (LAP), triglyceride-glucose index adjusted for waist-to-height ratio (TyG-WHtR), coronary risk (CR), visceral adiposity index (VAI) and abdominal volume index (AVI). For MetS prediction, the most influential features were VAI, LAP, waist triglyceride index (WTI), Very low-density cholesterol (VLDLC) and TyG-WHtR. Majority voting ensemble classifier demonstrated superior performance in predicting pre-MetS (AUC = 0.79) and MetS (AUC = 0.87).

**Conclusion:**

Identifying these risk factors reveals the complex interplay between visceral adiposity and metabolic dysregulation in African populations, enabling early detection and treatment. Ethical integration of ML algorithms in clinical decision-making can streamline identification of high-risk individuals, optimize resource allocation, and enable precise, tailored interventions.

**Supplementary Information:**

The online version contains supplementary material available at 10.1007/s40200-024-01491-7.

## Introduction

Affecting a substantial number of individuals and exerting profound pressure on modern healthcare, cardiometabolic diseases including type 2 diabetes mellitus (T2DM) have emerged as a formidable challenge, contributing to a significant health burden worldwide. Its subtle grip goes beyond a mere diagnosis, intruding into lives with the threat of long-term micro- and macro-vascular complications [1]. From retinopathy and nephropathy to coronary artery, cerebral disease, and peripheral artery disease, T2DM remains a serious condition that drives morbidity and hastens premature death [2]. This alarming reality is particularly pronounced in low- and middle-income countries where the burden is heavily felt [2]. Propelling this high prevalence of T2DM is metabolic syndrome (MetS), characterised by elevated blood pressure, dyslipidemia, elevated fasting glucose, and central or abdominal obesity [3–6]. Estimates indicate that MetS affects 20–25% of the global population, and individuals with MetS face a fivefold increased risk of developing T2DM [7, 8] which is expected to increase to 50% by 2030 [8].

In Ghana, several studies [3, 9–11] conducted in different regions have reported a higher incidence of MetS among individuals, with rates ranging from 24 to 78.8%. While the exact cause of MetS remain unclear, visceral adiposity is widely recognized as a significant contributing factor [12, 13]. Conventional methods for assessing visceral adiposity as MetS index include magnetic resonance imaging (MRI) and computed tomography (CT), which are sensitive but expensive and cumbersome [14–17]. Simpler methods, such as body mass index (BMI), waist circumference (WC), weight-to-height ratio (WHtR), and waist-to-hip ratio (WHR), are widely used but cannot differentiate between muscle mass from fat [18–22]. Recent studies have explored new indices like the waist triglyceride index (WTI), visceral adiposity index (VAI), triglyceride and glucose index (TyG), and lipid accumulation product (LAP) for better accuracy [23–24]. Although these indices have shown potential in various populations, their effectiveness in predicting MetS among Ghanaian patients with T2DM is yet to be established.

Statistical methods are frequently utilised to identify MetS risk factors, with risk ratio with risk ratio being a common approach. However, machine learning (ML) techniques are increasing seen as promising techniques due to their robust capability to analyse healthcare data. ML techniques have demonstrated superior performances compared to traditional statistical modelling approach, enabling the prediction of future trends and behaviour, which allows for proactive measures to prevent adverse outcomes or make informed decisions [25–26]. With advancements in big data analytics and data mining, a wide range of both structured and unstructured medical data can be leveraged to create predictive models. This approach stimulates a ‘prevention-focused’ care paradigm, as opposed to a traditional ‘intervention-based’ care approach [26–29]. ML algorithms represent a promising avenue for enhancing risk prediction and stratification in T2DM and related metabolic disorders. These algorithms can simultaneously analyse extensive datasets including demographic, anthropometric, biochemical, genetic, and lifestyle factors [30–31]. As a result, they possess the ability to accurately estimate individual risk profiles and capture complex interrelationships. Furthermore, ML algorithms dynamically learn from evolving data, which is particularly relevant in settings where the diabetes epidemic and associated risk factors are in constant flux. This adaptability allows for the development of specific-populations ML models. Utilising ML techniques, non-invasive indicators can be employed to predict MetS, enabling early diagnosis even in regions with limited medical conditions [29–32]. While numerous studies have applied ML techniques to predict MetS and enhance diagnostic accuracy, these studies come predominantly from advance countries [33]. There is a lack of similar research from Sub-Saharan African countries including Ghana, where MetS prevalence among T2DM is significant.

In this study, we leveraged ML techniques to develop an effective predictive model for MetS among individuals with T2DM. We incorporated biochemical parameters and novel anthropometric indices into the model. Notably, we included pre-metabolic syndrome (pre-MetS) indicators in the predictive framework, enabling the identification of patients most likely to benefit from early management strategies. This proactive approach aims to mitigate the progression of metabolic abnormalities and associated comorbidities, such as cardiovascular disease and chronic kidney disease, ultimately improving overall health outcomes and quality of life for individuals with diabetes.

## Materials and methods

### Data acquisition and population

Four de-identified datasets were retrospectively obtained from separate cohort studies investigating MetS in T2DM in Ghana. These datasets originated from various healthcare facilities: Komfo Anokye Teaching Hospital (KATH; *n* = 282) and Ejisu Government Hospital (EGH: *n* = 242) in the Ashanti region, Effia Nkwanta Regional Hospital (ENRH: *n* = 196) in the Western region, and Begoro District Hospital (BDH: *n* = 387) in the Eastern region. The four cohort studies (KATH, EGH, ENRH and BDH) share many similarities in target population, inclusion criteria, sampling design, data collection methods, cohort characteristics (including age, education level, occupational status and disease duration). Details on the description and comparability of these cohort studies are summarised in Supplemented Table 1. Data was collected about T2DM patients through questionnaires, health records and lab samples. Due to the similarities, combining the data from these studies was feasible. But difference in how some variables were measured or recorded necessitated data harmonisation [34]. This harmonisation focused onT2DM data from questionnaires, blood pressure measurements, anthropometrics and clinical measurements. Study protocols, standard operating procedures, questionnaires published papers, were reviewed and discussion between research team of KATH, EGH, ENRH and BDH data were held to understand the level of data heterogeneity and management across studies.

We selected variables relevant to our research objectives for harmonisation, evaluating whether they were completely or partially identical in the construct measured, question asked, response options, measurement scale, frequency, and coding features. Variables with an exact match in all these aspects were deemed completely matching. Partially matching variables had the same construct but differed in frequency response options or coding. Completely unmatching variables with lack the construct in one of the datasets or used different measurement scales (Supplementary Table 2). Quality control procedures were employed to test and describe the quality of harmonised data. Descriptive statistics of each harmonised variable were conducted in each dataset to assess the consistency and distribution of participants (Supplementary Table 3). The final harmonised data set was then used to address our research objectives.

The datasets encompassed a total of 1107 participants and included information on T2DM patients’ sociodemographic characteristics, anthropometric and hemodynamic indices, and various clinical biochemical parameters. We extracted as many features as possible to identify informative feature variables for diagnosis MetS. A comprehensive overview of all features within each dataset based on their respective types is provided in Table 1.

**Table 1:** Features in the data set according to their type

Type	Features
Patient demographic information	Age, Sex, Marital status, Education level, Occupation
Conventional anthropometric indices	Height, Weight, WC, HC, WHR, WHtR, BMI, BAI, CI, AVI
Novel anthropometric indices	VAI, WTI, LAP, TyG, TyG-BMI, TyG-WC, TyG-WHtR, TyG-WHR
Hemodynamic indices	SBP (mmHg), DBP (mmHg)
Biochemical parameters	HBA1c, FBS (mmol/L), TCHOL (mmol/L), TG (mmol/L), HDL-C (mmol/L), LDL-C (mmol/L), CR, VLDL-C (mmol/L).

WC Waist Circumference, HC: Hip Circumference, WHR: Waist-to-Hip Ratio, WHtR: Wait-to-Height ratio, BMI: Body Mass Index, CI: Conicity Index, AVI: Abdominal Volume Index, VAI: Visceral Adiposity Index, WTI: Waist Triglyceride Index, LAP: Lipid Accumulation Product, TyG: Triglyceride and Glucose Index, TyG-BMI: Triglyceride Glucose-Body Mass Index Ratio, TyG-WC, Triglyceride Glucose-Waist Circumference Ratio, TyG-WHtR: Triglyceride Glucose-Waist-to-Height Ratio, TyG-WHR: Triglyceride Glucose-Waist-to-Hip Ratio, SBP: Systolic Blood Pressure, DBP: Diastolic Blood Pressure TG: Triglycerides, TCHOL, Total cholesterol, HDLC, High-density lipoprotein cholesterol, LDLC, Low-density lipoprotein cholesterol, CR: Coronary risk; VLDLC: Very low-density lipoprotein cholesterol, Fasting blood sugar; HbA1C, Glycated haemoglobin

### Blood pressure measurement

Blood pressure was measured by qualified nurses using a mercury sphygmomanometer and stethoscope. The recommendation of the American Heart Association (AHA) was used to take measurements from the upper left arm after participants had sat for more than 5 min. The average value for the two measurements (with a 5-minute break interval between measurements) was recorded to the nearest 2.0 mmHg.

### Anthropometric and clinical measurements

The height of subjects was measured to the nearest 0.1 cm without shoes and weight was also measured to the nearest 0.1 kg with participants in light clothing. A bathroom scale (Zhongshan Camry Electronic Co., Ltd., Guangdong, China) was used to weigh the participants and their height was measured with a stadiometer (Seca 213 mobile stadiometer, Germany) while they stood upright with their backs straight, heels together, and their feet slightly apart at a 60° angle. Waist circumference (to the nearest 0.1 cm) was measured with a Gulick II spring-loaded measuring tape (Gay Mills, WI) halfway between the inferior angle of the ribs and the supra iliac crest. The hip circumference was measured at the widest diameter around the gluteal protuberance to the nearest 0.1 cm.

The novel anthropometric indices WTI, VAI, LAP, and TyG were calculated according to the following formulas:

WTI = Ln [TG (mg/dl) x WC (cm)/2] [23].

VAI = [WC (cm)/39.68 + (1.88 x BMI (kg/m^2^))] x (TG (mmol/L)/1.03) x (1.31/HDL-C (mmol/L) for male and [WC (cm)/36.58 + (1.89 x BMI (kg/m^2^))] x (TG (mmol/L)/0.89) x (1.52/HDL-C (mmol/L) [24].

LAP = [WC (cm)– 65] x TG (mmol/L) for male and [WC (cm)– 58] x TG (mmol/L) for female [35, 36].

TyG = Ln [TG (mg/dl) x fasting blood glucose (mg/dl) / 2] [37].

### Blood sampling and biochemical analysis

A volume of five (5) millilitres (ml) of venous blood samples was collected after an overnight fast; 4 ml was dispensed into a serum separator tube and 1 ml into fluoride-oxalate tubes. After centrifugation at 3000 rpm for 15 min, the serum and plasma were stored at − 80 °C until assayed. Parameters included Fasting plasma glucose (FPG), glycated haemoglobin (HbA1C), total cholesterol (TC), triglycerides (TG), and high-density lipoprotein (HDL) cholesterol were assayed using the COBAS INTEGRA^(R)^ 400 *plus* Automated Chemistry Analyzer. The protocol for the determination of the parameters was as indicated in the manufacturer’s instructions (Fortress Diagnostics Limited, Unit 2 C, Antrim Technology Park, Antrim BT41 1QS, United Kingdom). Low-density lipoprotein cholesterol (LDL-c) was calculated using the Friedewald formula [LDL] = [TC] − [HDL] − [TG]/2.2(1) mmol/L [38].

### Definition of metabolic syndrome and pre-metabolic syndrome (pre-MetS)

Metabolic syndrome was defined by the presence of three or more of the five risk factors based on the International Diabetes Federation (IDF) criteria [39], which involve the presence of central obesity (defined as waist circumference but can be assumed if BMI > 30 kg/m^2^) with ethnicity-specific values* plus any two of the following: Triglycerides 150 mg/dl or greater, HDL-cholesterol < 40 mg/dl in men and < 50 mg/dl in women or ongoing therapy for dyslipidaemia; BP 130/85 mmHg or greater or ongoing therapy for hypertension; fasting glucose 100 mg/dl or greater or ongoing diabetes therapy. Pre-metabolic syndrome (pre-MetS) was defined as having no less than two components of MetS but did not meet the criteria for the diagnosis of MetS [40].

### Basic statistics

Statistical analysis was performed in R version 4.3.3 [41]. Categorical data were presented as frequencies (proportions). The normality of continuous variables was tested by Kolmogorov–Smirnov. Non-parametric data were presented as median (interquartile range). The Kruskal-Wallis rank sum test was used to compare feature with continuous data using the ‘dplyr’ package [42] and Chi-Square test for categorical data among the different groups of metabolic components using the basic R ‘chiseq.test()’ function. All statistical results obtained were considered at a significant value of *p* < 0.05.

### Data pre-processing

Raw data were pre-processed to clean, format, and organise the information. From the initial 1,107 records and 33 attributes, patient records and attributes with ≥ 1.0% missing information were excluded to prevent potential biases and inaccuracies that can arise from extensive imputation. This conservative 1% threshold ensured a robust final dataset less likely to introduce biases into the machine learning model. Also, attributes that form part of the diagnostic criteria of MetS were removed from the prediction algorithm. The resulting dataset comprised 919 participants with 21 feature variables, which was sufficient for data analysis. Among these participants, 309 (33.5%) had MetS, 238 (25.9%) had pre-MetS, and 372 (40.5%) had no MetS. Further data refinement included checking and removing outlying values in numeric variables, addressing missing numeric values through median imputation, and creating dummy variables for the categorical ‘sex’ variable (female = “0”, male = “1”). The processed data underwent checks for zero and near-zero variance feature variables [43], confirming that all feature variables had more than one unique value.

### Feature selection and classification

Feature selection optimises predictive modelling by reducing input variables, enhancing computational efficiency, and improving model performance. Statistical-based feature selection methods evaluate relationships between input and target variables, retaining the most influential variables [44–45]. The models in our study focussed on differentiating between the absence of Mets vs. pre-MetS and MetS. We employed the recursive feature elimination (RFE) with cross-validation (RFECV) algorithm [46] and the Least Absolute Shrinkage and Selection Operator (LASSO) [47] feature selection method for feature selection. RFE is a wrapper-type feature selection approach that necessitates an external estimator or ML algorithm to assign coefficients or ranks to features and selects the most important ones based on the assigned coefficient or ranks [48]. Hence, the decision tree classifier (DTC) algorithm was utilised within the core of the RFE method, employing 5-fold cross-validation loops to automatically determine the optimal number of features. Subsequently, a decision tree was fitted on the selected features to achieve classification accuracy.

LASSO feature selection, on the other hand, is an embedded method where feature selection occurs during model fitting [48]. Consequently, the LASSO regression algorithm was used for this purpose. For MetS, the RFCEV-DTC identified 20 feature variables with an accuracy of 80.7% (± 0.03) while lasso regression identified 17 feature variables with 17 features overlapping between the two methods. For pre-MetS, RFCEV-DTC found 19 feature variables with an accuracy of 68.1 (± 0.04) and lasso regression resulted in 17 feature variables, sharing 17 common features. Additionally, non-parametric Spearman correlation was performed on numerical selected feature variables for Pre-MetS and MetS with a cut-off of 0.9. None of the features were identified to be highly correlated.

Finally, we used the BORUTA method [49] to validate the results obtained from RFCEV and Lasso regression methods based on the common features for both pre-MetS and MetS. As a wrapper method, the Boruta algorithm leverages random forest classification for feature relevance estimation [49]. We performed the Boruta algorithm to confirm the final list of features deemed most important setting the ‘doTrace’ argument to 3, which was then used for developing ML models.

### Machine learning algorithms

We employed six different ML algorithms in our study. Two traditional models—Logistic Regression for Classification (LR) [50] and Gaussian Naive Bayes classification (GNB) [51] —were complemented by three advanced models: k-nearest neighbour classification (KNN) [52], Support Vector Machine (SVM) [53], Gradient Boosting for Classification (GBC) and Ensemble Voting [54]. Logistic regression, a commonly used method in ML for classification tasks, models outcomes of the probabilities based on predictor variables without requiring a linear relationship between them [55]. KNN, with its simple implementation, is robust, even when classes are not linearly separable [56–57]. GNB uses Bayes’ theorem to categorize observations into predefined classes based on predictor variables. It assumes independence between predictor variables for each class [58]. SVM constructs hyperplane multi-dimensional spaces for classification, regression, or outlier detection. SVM excels when there is a clear separation margin between classes, even in high-dimensional spaces [59]. GBC builds an additive model in a forward stage-wise fashion that allows for the optimization of arbitrary differentiable loss functions. In each stage *n* classes regression trees are fit on the negative gradient of the loss function [60].

Ensemble methods integrate multiple individual classifiers to create a meta-classifier, often yielding superior generalization performance compared to single classifiers. We employed widely-used ensemble techniques based on the majority voting (MV) principle. In MV, the final classification decision is determined by the most frequent class prediction among the cinstiuent classifiers [61, 62]. We formulated our models in Python 3.8.0, ultilizing the library sklearn 0.23.2 [63]. The data was randomly split into 80% training and 20% test sets, maintaining an equal MetS case rate by stratified sampling with 100 random repeats. The same data-splitting approach was applied to pre-MetS cases. Our ML models were aimed to identify patterns in input data (selected BORUTA features) that discriminate among MetS groups (No MetS, pre- MetS and MetS).

We assessed the performance of 5 different individual classifiers —LR, GNB, SVM, KNN and GBC, and their ensemble (MV) on the T2DM MetS dataset. We used a cross-validation strategy with 5 splits, repeated 3 times to ensure stratified sampling to maintain the proportion of classes in each fold. The different classifiers were defined with specific hyperparameters, and each classifier was combined with a “StandardScaler” for preprocessing with a “Pipeline” to ensure all classifiers received normalised input data. We then combined the classifiers into an Ensemble vote classifier using the Majority Vote classifier. Here, each classifier was given an equal weight of ‘1’, indicating that each classifier’s vote had the same influence on the final decision. Each individual classifier (LR, GNB, SVM, KNN and GBC) and the Majority voting classifier were evaluated using 10-fold cross-validation on the training data.

**Fig. 1:**
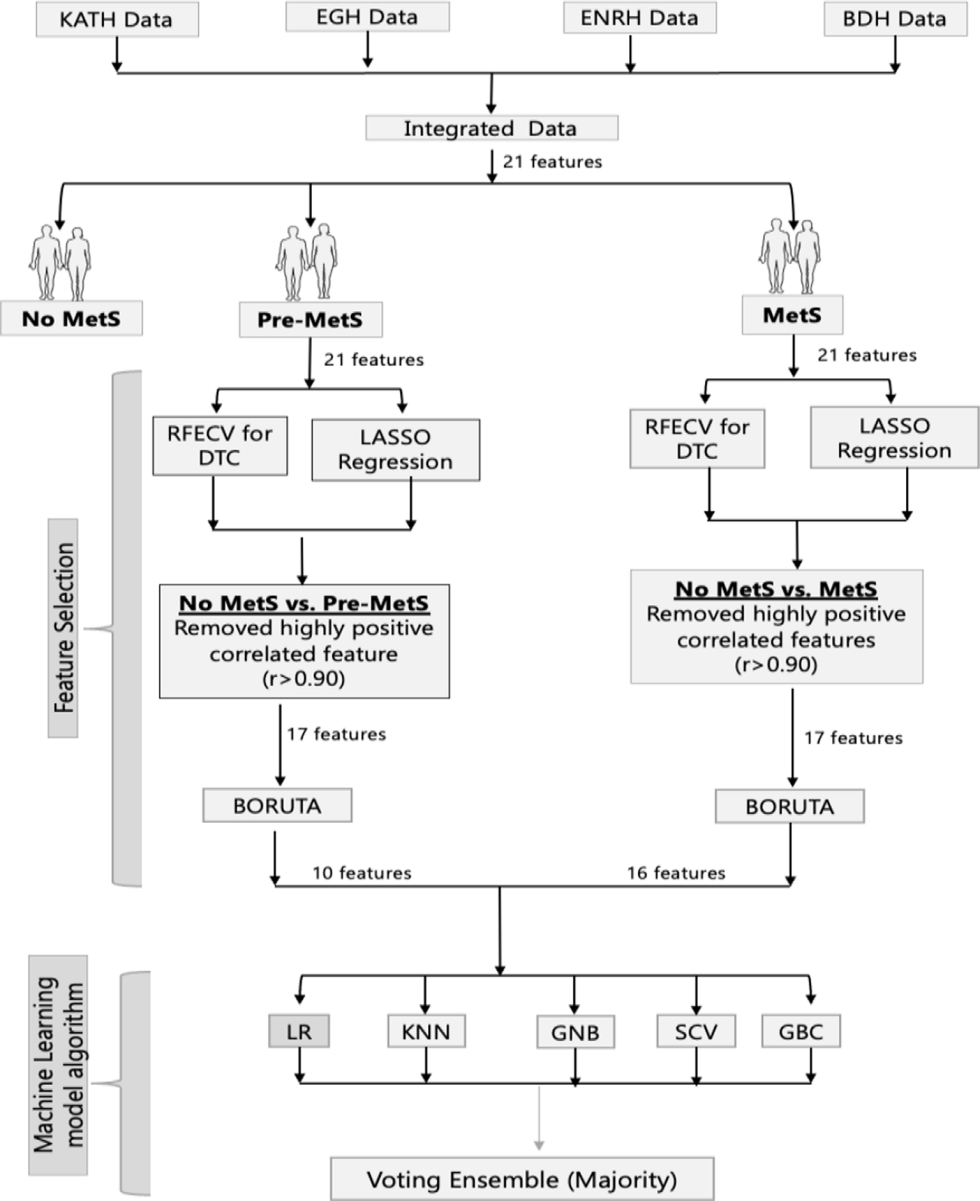
Machine learning pipeline for selection of indices for pre-metabolic syndrome and metabolic syndrome

The trained models were then used to make predictions on the test data. Parameters were tuned using the ParameterGrid function of the model Selection class provided by sklearn, with an area under the receiver operating characteristic curve (ROC-AUC) as the evaluation criterion. ROC curves were generated from the test data set to ensure that the MajorityVoteClassifier generalised well with unseen data to provide an unbiased estimate of the generalisation performance of the classifier system. The parameter tuners used as input to each ML model are listed in Table 2. The summary of the data harmonisation and analytical workflow are presented in Fig. 1.

**Table 2:** Parameters stunning setting for the ML classification

ML Classifiers	Grid of parameters
Logistic Regression (LR)	penalty=’12’,
	C = 0.1,
	solver=’liblinear’,
	random_state = 1
K-Neighbours Classifier (KNN)	n_neighbors = 11, weights = “distance”,
	*p* = 2, metric=’manhattan’
Gaussian Naïve Bayes (GNB)	var_smoothing = 0.0002
Support Vector Machine (SVM),	C = 100, random_state = 1,
	gamma = 0.01, probability = True
EnsembleVoteClassifier	(clfs=[LR, KNN, GNB, SVM], weights=[1,1,1,1,1])

Cross validation = RepeatedStratifiedKFold(n_splits = 5, n_repeats = 3, random_state = 1)

### Models performance evaluation

The performance of each model in predicting outcomes was evaluated using calibration and discrimination metrics in the test data set. Calibration was measured using the Brier score, which ranges from 0 to 1 with lower scores indicating better calibration [64]. Discrimination was evaluated using the ROC-AUC metric. Additionally, precision, F1-score, and accuracy were assessed in the test set. Precision represents the ratio of correctly predicted positive observations to all actual positives. F1-score on the other hand, is the weighted mean of precision and recall. Finally, accuracy signifies the ratio of correctly predicted observations to the total observations.

### Data visualisation

Data visualisation plays a crucial role in research, offering an intuitive means to identify data trends or intricate insights that might otherwise be challenging to discern. Heatmaps represent a widely used method for visualising high-dimensional data with multiple variables. We employed a heatmap with unsupervised hierarchical clustering to visualise the associations of metabolic phenotype clusters with MetS Progression. Furthermore, principal component analysis (PCA) was performed to visualize the distribution of subgroupings among components of MetS and feature variables using the two most dominant PCA components [65–66].

## Results

### Baseline characteristics

Detailed baseline characteristics of the study participants stratified by MetS status (no MetS, pre-MetS, and MetS) are presented in Table 3. Significant differences were observed across various anthropometric, biochemical, and clinical parameters among the three groups. Notably, individuals with MetS exhibited higher values for weight, WC, HC, WHR, WHtR, BMI, BAI, CI, VAI, WTI, LAP), TyG, TyG-BMI, TyG-WC, TyG-WHtR, SBP, DBP, FBS, TCHOL, TG, LDL-C, CR, and VLDL-C compared to the no MetS and pre-MetS groups. Conversely, individuals with MetS had lower levels of HDL-C compared to the other two groups [Table 3].

**Table 3:** Characteristics of patients based on metabolic syndrome status

Variables	Total (*n* = 919)	No MetS (*n* = 372)	Pre-MetS (*n* = 238)	MetS (*n* = 309)	*P*-value
Age (years)	53.2(52.5–53.8)	52.3(51.4–53.3)	53.1(51.8–54.4)	54.2(53.1–55.3)	0.057
Sex (m/f)	341/578	164/208	78/160	99/210	0.001
Height (m)	1.62(1.57–1.68)	1.63(1.57–1.69)	1.63(1.63–1.68)	1.63(1.61–166)	0.130
Weight (kg)	71.6(70.7–72.5)	68.3(66.8–69.7)	72.5(70.8–74.2)	74.9(73.5–76.3)	< 0.001
WC (cm)	93.5(92.7–94.3)	89.4(88.0–90.7)	94.0(92.6–95.5)	97.9(96.7–99.2)	< 0.001
HC (cm)	102.6(101.8–103.5)	100.6(99.2–101.9)	102.8(101.3–104.4)	105.0(103.8–106.3)	< 0.001
WHR	0.92(0.91–0.94)	0.89(0.88–0.91)	0.92(0.90–0.94)	0.93(0.92–0.94)	< 0.001
WHtR	0.57(0.56–0.58)	0.55(0.54–0.56)	0.58(0.57–0.59)	0.61(0.59–0.61)	< 0.001
BMI (kg/m^2^)	27.2(26.8–28.3)	25.8(25.2–26.3)	27.5(26.8–28.3)	28.6(28.0–29.1)	< 0.001
BAI	31.7(31.2–32.2)	30.5(29.8–31.2)	31.8(30.9–32.8)	33.1(32.4–33.9)	< 0.001
CI	1.29(1.28–1.31)	1.27(1.26–1.29)	1.29(1.28–1.31)	1.33(1.31–1.34)	< 0.001
AVI	17.9(17.6–18.2)	16.5(16.0–17.0)	18.1(17.6–18.7)	19.5(19.0–19.9)	< 0.001
VAI	1.76(1.66–1.86)	1.26(1.17–1.34)	1.60(1.44–1.76)	2.48(2.24–2.72)	< 0.001
WTI	7.02(6.99–7.06)	6.80(6.75–6.85)	7.01(6.90–7.07)	7.31(7.26–7.36)	< 0.001
LAP	44.1(42.0–46.3)	30.9(28.2–33.6)	42.9(39.0–46.8)	61.1(57.3–64.9)	< 0.001
TyG	7.54(7.49–7.58)	7.33(7.27–7.39)	7.53(7.45–7.61)	7.80(7.72–7.87)	< 0.001
TyG-BMI	205.1(202.2–213.1)	189.0(184.7–193.4)	207(201.9–213.1)	222.7(218.2–227.3)	< 0.001
TyG-WC	704.9(697.6–712.3)	654.4(643.3–665.5)	708.0(695.1–720.9)	763.5(752.2–774.6)	< 0.001
TyG-WHtR	4.34(4.29–4.40)	4.02(3.95–4.09)	4.37(4.28–4.45)	4.72(4.65–4.80)	< 0.001
TyG-WHR	6.91(6.83–6.97)	6.57(6.45–6.68)	6.94(6.79–7.10)	6.91(6.83–6.97)	< 0.001
SBP (mmHg)	130.2(128.8–131.5)	118(116.9–119.7)	130(127.133.1)	144.1(142.1–146.2)	< 0.001
DBP (mmHg)	78.4(77.6–79.0)	71.3(70.5–72.1)	77.8(76.5–79.2)	87.4(86.2–88.7)	< 0.001
FBS (mmol/L)	9.36(9.09–9.64)	8.93(8.57–9.30)	9.54(8.90–10.10)	9.73(9.21–10.26)	0.034
TCHOL (mmol/L)	5.29(5.19–5.38)	5.05(4.91–5.19)	5.28(5.08–5.47)	5.57(5.40–5.74)	< 0.001
TG (mmol/L)	1.51(1.46–1.56)	1.20(1.18–1.32)	1.45(1.36–1.53)	1.85(1.76–1.94)	< 0.001
HDL-C (mmol/L)	1.30(1.28–1.33)	1.41(1.37–1.45)	1.32(1.27–1.38)	1.16(1.12–1.20)	< 0.001
LDL-C (mmol/L)	3.47(3.38–3.56)	3.17(3.04–3.30)	3.49(3.31–3.67)	3.81(3.65–3.97)	< 0.001
CR	4.44(4.30–4.57)	3.85(3.68–4.02)	4.28(4.05–5.53)	5.26(4.98–5.53)	< 0.001
VLDL-C (mmol/L)	0.68(0.66–0.71)	0.57(0.54–0.60)	0.66(0.62–0.88)	0.84(0.80–0.88)	< 0.001

WC Waist Circumference, HC: Hip Circumference, WHR: Waist-to-Hip Ratio, WHtR: Wait-to-Height ratio, BMI: Body Mass Index, CI: Conicity Index, AVI: Abdominal Volume Index, VAI: Visceral Adiposity Index, WTI: Waist Triglyceride Index, LAP: Lipid Accumulation Product, TyG: Triglyceride and Glucose Index, TyG-BMI: Triglyceride Glucose-Body Mass Index Ratio, TyG-WC, Triglyceride Glucose-Waist Circumference Ratio, TyG-WHtR: Triglyceride Glucose-Waist-to-Height Ratio, TyG-WHR: Triglyceride Glucose-Waist-to-Hip Ratio, SBP: Systolic Blood Pressure, DBP: Diastolic Blood Pressure TG: Triglycerides, TCHOL, Total cholesterol, HDLC, High-density lipoprotein cholesterol, LDLC, Low-density lipoprotein cholesterol, CR: Coronary risk; VLDLC: Very low-density lipoprotein cholesterol, Fasting blood sugar, *P* < 0.05 considered statistically significant

### Associations between baseline characteristics across the MetS status

**Fig. 2:**
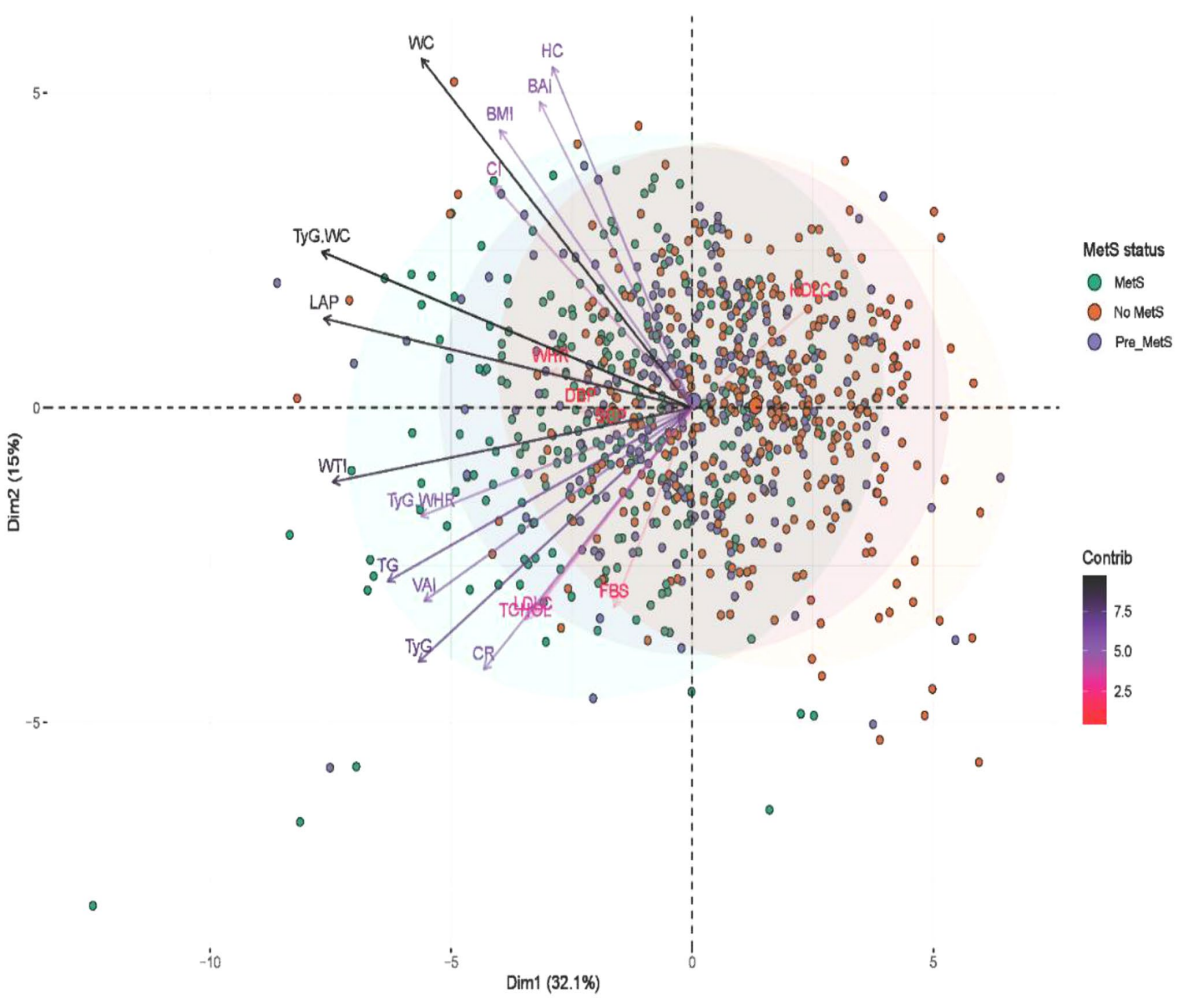
PCA Biplot of principal component axes and MetS statuses among T2DM

In assessing the feature importance and relationships between the study variables relative to the MetS status, Principal Component Analysis (PCA) revealed significant overlap among the metabolic profiles associated with different metabolic syndrome (MetS) status among individuals with T2DM. Figure 2 suggests that metabolic parameters analysed highlight the metabolic dysregulation accompanying the progression from a healthy state to pre-MetS and overt MetS. The length of the vector for the variables indicates the importance of the variable in discriminating between MetS status. For example, WC, TyG-WC, LAP, WTI, and TyG were highly distinctive for the MetS group compared to the other two groups. The angle between the vectors indicates the nature of the relationship, angles less than 90 degrees indicate positive relations, angles approximating 180 degrees indicate negative relations, and angles close to 90 degrees indicate no relationship [26]. For example, there are strong positive relationships between TyG and CR and TyG-WC and LAP [Fig. 2].

### Metabolic phenotype clusters associated with MetS progression

**Fig. 3:**
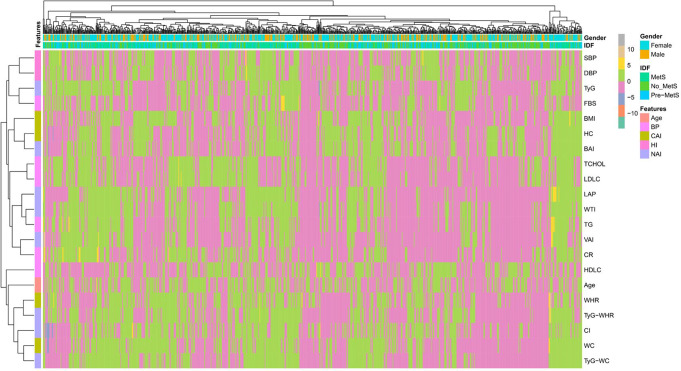
Heatmap displaying the correlation matrix across demographic, anthropometric, and clinical features in the study cohort. The x and y axes indicate the features examined, with matching row and column labels. The coloured cells represent the Pearson correlation coefficients, with the colour gradient ranging from red (negative correlation) to blue (positive correlation); deeper shades indicate stronger correlations. Notable patterns include strong positive correlations of gender with IDF, SBP, and TyG; intricate correlation patterns for IDF, showing positive associations with pre-metabolic conditions and negative links to overt metabolic syndrome; clustering of obesogenic markers (BMI, BAI, VAI), dyslipidaemia indicators (CHOL, HDL-C, LDL-C), and dysglycemia/insulin resistance markers (FBS, TCHOL, TyG); and differential age correlations with elevated blood pressure/dyslipidaemia versus lower obesity indices

Hierarchical clustering analysis [Fig. 3] was performed to investigate the metabolic profiles among individuals based on their MetS status. The heatmap reveals distinct clusters corresponding to the no MetS, pre-MetS, and MetS groups, further reinforcing the presence of distinct metabolic phenotypes associated with MetS progression. The analysis supports the notion of a continuum of metabolic disturbances, with pre-MetS representing an intermediate state between health and overt MetS.

Gender exhibits strong positive correlations with a subset of features, including SBP, and TyG. This suggests that gender plays a key role in modulating these metabolic and physiological parameters within our study population. Clustering of related features is also evident. For instance, obesogenic indicators such as BMI, BAI, VAI, and indicators of dyslipidaemia (CHOL, HDL-C, LDL-C) form a tight cluster of positive inter-correlations. Similarly, markers of dysglycemia and insulin resistance (FBS, TCHOL, TyG) are highly correlated with one another. These observed clusters align with established biological underpinnings linking anthropometric measures, lipid metabolism, and glucose homeostasis in metabolic disease pathogenesis.

Age exhibits a differential correlation pattern– positively associated with blood pressure and lipid markers such as TG, while negatively linked to obesity indices (e.g., BMI) and visceral adiposity. This may reflect contrasting age-related effects on body fat distribution versus cardiometabolic risk biomarkers. The gender-metabolic associations revealed, coupled with the differential correlation structure of age, and clustered metabolic risk factors, provide discrete analytical perspectives to define metabolic health trajectories across demographic and clinical boundaries in this study cohort [Fig. 3].

### Feature selection

**Fig. 4:**
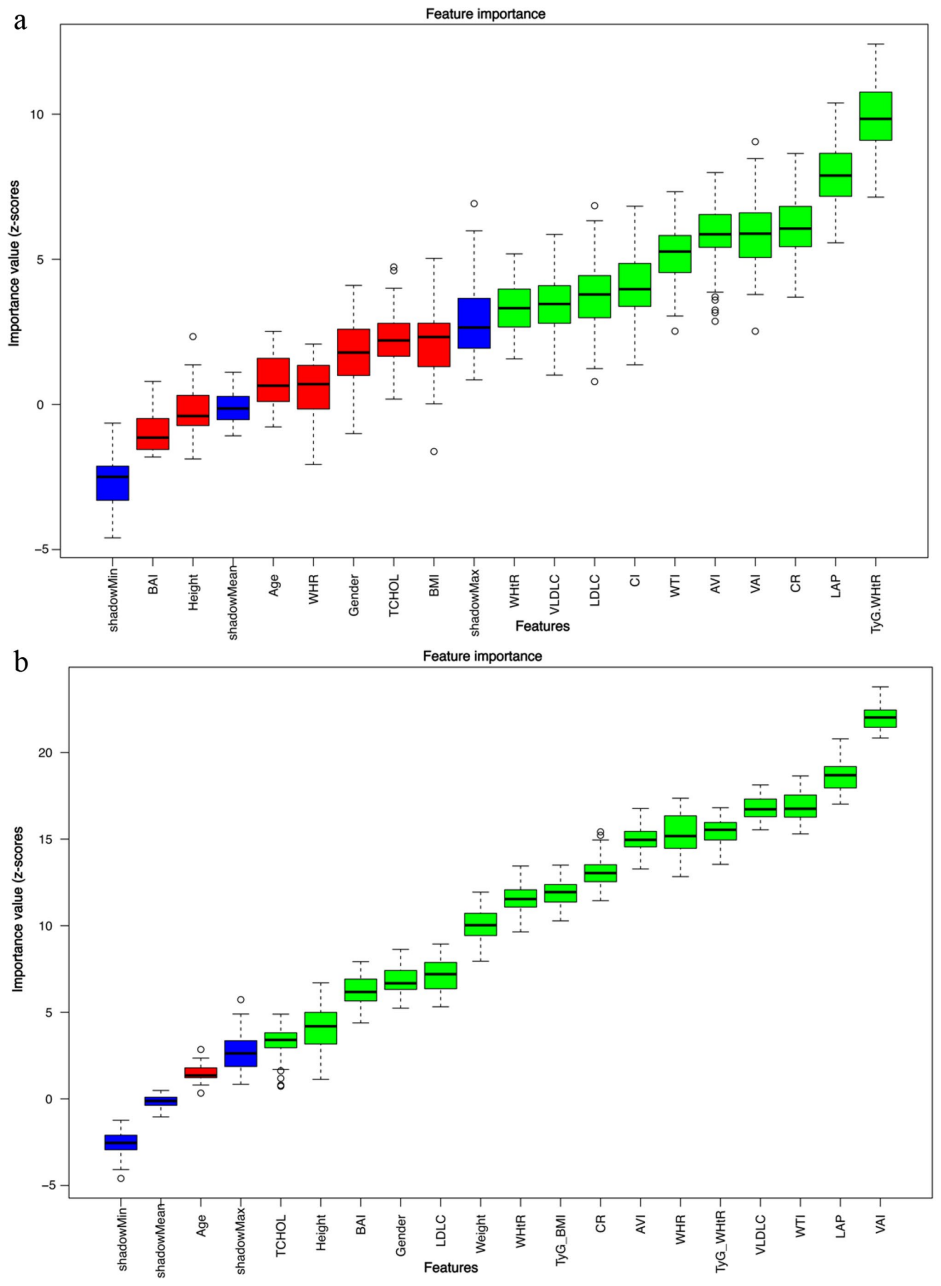
Feature selection importance graphs using the BORUTA algorithm for pre-MetS AND and MetS. Blue boxplots correspond to minimal, average, and maximum Z scores of a shadow attribute. Red and green plots represent Z scores of respectively rejected and confirmed features

The BORUTA algorithm was employed to identify the most important features for predicting pre-MetS and MetS status. For pre-MetS prediction, the five top-ranked features were the TyG-WHtR, LAP, CR, VAI AVI [Fig. 4a]. Notably, for MetS prediction, the five most influential features were VAI, LAP, WTI, VLDLC, AND TyG-WHtR [Fig. 4b].

### Classification performance of ML models

**Fig. 5:**
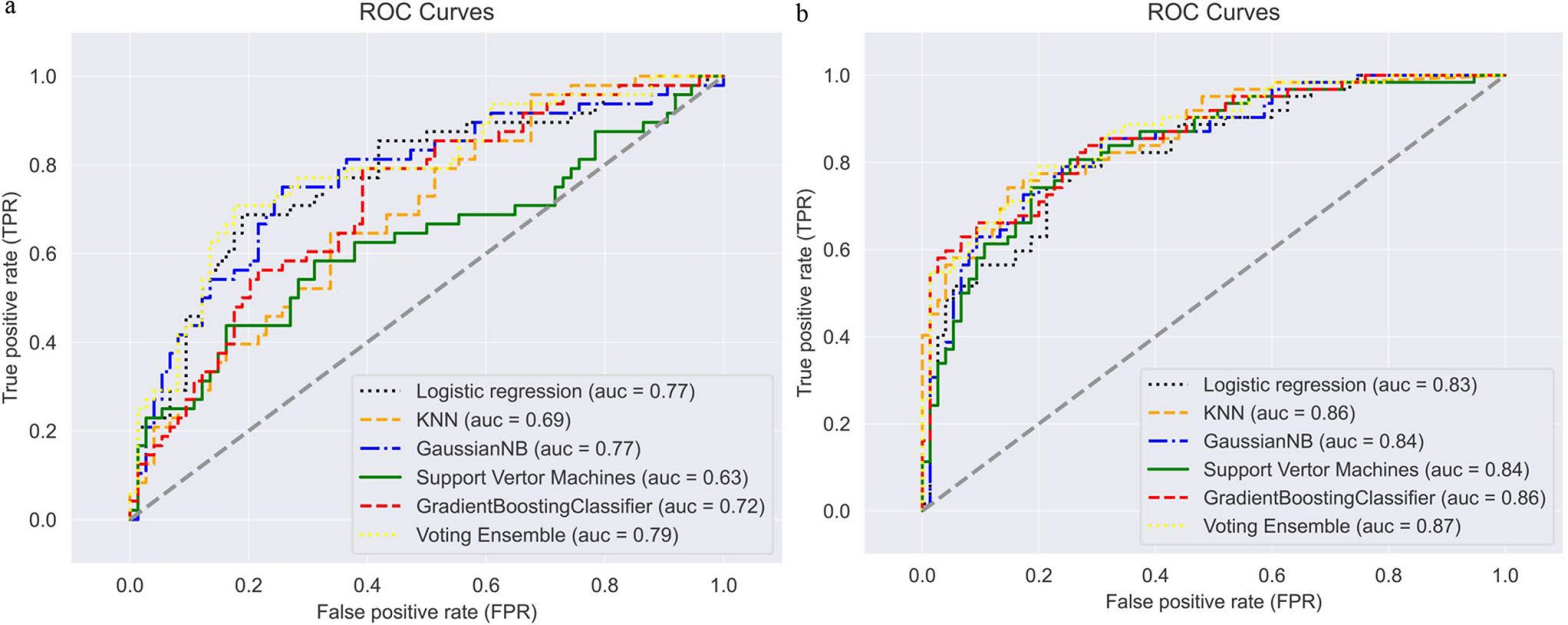
Receiver operating characteristic curves of ML models for predicting **a**) pre-MetS and **b**) MetS among T2DM patients

The BORUTA algorithms identified 11 and 14 significant features for pre-MetS and MetS respectively and these were used as inputs in the ML models. Receiver operating characteristic (ROC) curves were constructed to evaluate the performance of various machine learning models in predicting pre-MetS and MetS among individuals with T2DM [Fig. 5]. The voting ensemble (VE) model demonstrated superior predictive performance, exhibiting the highest ROC-AUC values for both pre-MetS (AUC = 0.79) and MetS (AUC = 0.87) prediction tasks. The ensemble classifier performed better on the test dataset than individual algorithms.

Table 3 presents a comprehensive evaluation of the ML algorithms using metrics like accuracy, specificity, sensitivity, precision, and F1 scores. The VE model, in line with ROC curve analysis, demonstrated the highest accuracy of pre-MetS prediction at 73.0% followed by GNB at 72.9%. The LR had the lowest accuracy at 63.9%. VE also leads in MetS prediction with an accuracy of 78.1% with GNB close behind at 77.8%) and the least being LR (73.0%) [Table 4]. These findings underscore the superior performance of the ensemble-based method in accurately identifying individuals at risk of developing pre-MetS and MetS.

**Table 4:** Metrics and performance comparison among ML algorithms for predicting the presence of Pre-MetS and MetS

Model	status	Accuracy	Specificity	Sensitivity	Precision	F1 scores	Brier loss
	Pre-MetS						
LR		0.705	0.901	0.395	0.730	0.513	0.202
KNN		0.639	0.797	0.395	0.558	0.463	0.212
GNB		0.729	0.770	0.667	0.653	0.659	0.192
SVC		0.655	0.837	0.375	0.600	0.461	0.232
GBC		0.656	0.840	0.396	0.594	0.475	0.207
VE		0.730	0.905	0.438	0.758	0.571	0.191
	**MetS**						
LR		0.730	0.813	0.629	0.736	0.678	0.170
KNN		0.744	0.720	0.774	0.696	0.732	0.150
GNB		0.778	0.827	0.709	0.771	0.739	0.195
SVC		0.766	0.787	0.741	0.741	0.732	0.164
GBC		0.773	0.787	0.661	0.803	0.726	0.148
VE		0.781	0.853	0.693	0.797	0.741	0.142

LR Logistic Regression for Classification, GNB: Gaussian Naive Baye: KNN: k-nearest neighbour classification, SVM: GBC: Gradient Boosting Classification, Support Vector Machine, VE: Voting Ensemble

## Discussion

The present study employed advanced ML techniques and novel anthropometric indices to develop robust predictive models for pre-MetS and MetS among individuals with T2DM. By identifying specific distinct risk factors associated with pre-MetS and MetS, we can develop personalized intervention approaches to curb underlying metabolic abnormalities. Our analysis identified key risk factors for both pre-MetS and MetS, with LAP, VAI, and TyG-WHtR emerging as common predictors for both conditions. Additionally, CR and AVI were found to be primary risk factors for pre-MetS, while WTI and VLDLC were identified as significant predictors specifically for MetS. Notably, these identified risk factors were not mutually exclusive, presenting in various combinations in individuals. However, distinct metabolic profiles were noted for different MetS statuses, corroborating previous findings of dysregulated metabolic pathways in pre-MetS and MetS [67–68]. The clear distinction of these profiles along the principal component axes suggests that the analysed metabolic parameters effectively captured the metabolic imbalances linked to MetS progression. Moreover, observed significant differences in anthropometric, biochemical, and clinical parameters across MetS groups are in line with the established MetS diagnostic criteria and the well-documented associations between obesity, insulin resistance, and metabolic perturbations [6]. These findings support the concept of a continuum of metabolism dysregulation [69–70], indicating that early detection and management of individuals in the pre-MetS stage could potentially prevent or delay progression to MetS and associated complications, underscoring the clinical relevance of the developed predictive models.

Our study demonstrates an approach that addresses a critical need for early identification of at-risk individuals, potentially before all MetS components reach diagnostic thresholds [71]. The synergistic effects captured by our indices provide additional predictive value beyond individual factor analysis, aligning with recent findings on the complex interplay of MetS components [72]. In resource-limited settings like Ghana, where comprehensive MetS screening may be challenging, our model offers a valuable tool for identifying patients requiring further evaluation [73]. By focusing on MetS rather than specific conditions like diabetes, our research maintains a broader scope with wider applicability in general health screenings. This approach enables the identification of individuals at earlier stages of metabolic dysfunction, facilitating timely lifestyle interventions. Recent studies have highlighted the importance of such early interventions in preventing the progression to diabetes and cardiovascular disease [74, 75]. Our model’s ability to predict MetS using a subset of easily obtainable measurements addresses the practical constraints often faced especially in low-resource healthcare settings [76].

The performance of the various machine learning algorithms in predicting MetS, a complex, multi-faceted condition, provides valuable insights into the application of AI in composite health outcome prediction. This aligns with the growing body of research on AI applications in public health [77]. When incorporated into clinical decision support systems, prediction tools such as those used in this study could aid healthcare professionals in initiating timely interventions. Due to the interplay of complex genetic and environmental factors contributing to these risk factors multifaceted approach is required to mitigate its detrimental effects. This may include dietary modifications such as adopting a low-calorie, low-fat diet coupled targeted exercise program designed to reduce visceral fat [78–79]. If lifestyle modifications are not enough to provide satisfactory results, pharmacological interventions, such as lipid-lowering agents (e.g., statins, fibrates) and anti-obesity medications, may also be considered for individuals with dyslipidaemia or refractory obesity [80–81].

We observed a superior performance of the majority voting classifier in predicting pre-MetS and MetS can be attributed to their ability to combine multiple weak learners, resulting in improved predictive power and robustness [82]. Our methodology was comparable with several other studies that employed ML techniques for predicting metabolic disorders. For instance, Tavares et al. [83], Avizohar et al. [84], Li et al. [85], Hu et al. [86] and Park et al. [87] utilized ML techniques including LR, linear discriminant analysis (LDA), KNN, decision trees, Light Gradient Boosting Machine (LGBM), Extreme Gradient Boosting, naïve Baye, respectively, to predict MetS. While these studies demonstrated high predictive accuracy, our approach incorporates novel anthropometric indices and pre-MetS indicators, which provide a more nuanced risk stratification. Moreover, the use of ensemble learning in our study enhanced the predictive performance compared to single-model approaches used in previous research. Voting ensemble leverages the collective wisdom of multiple base models, each contributing its unique strengths and weaknesses, to create a more powerful and accurate predictive algorithm. By aggregating the predictions of diverse base models, ensemble techniques can effectively capture complex patterns and nuances within the data, leading to enhanced predictive capabilities compared to individual models. The exceptional performance of the ensemble model highlights their suitability for the complex task of predicting pre-MetS and MetS, given the complex interactions among multiple risk factors and biomarkers involved [6, 67–68, 78, 79, 80, 88].

The predictive models developed in this study are poised to act as low-cost, non-invasive tools for identifying high-risk individuals who would benefit from targeted prevention and management interventions [26, 27]. These models leverage readily available clinical and demographic data, eliminating the need for expensive or invasive diagnostic procedures. This accessibility makes them particularly valuable in resource-limited settings or for large-scale screening programs. By facilitating early identification and control of cardiometabolic risk factors associated with MetS and its precursors, these models have the potential to significantly reduce the morbidity and mortality burden caused by diabetes-related complications [89]. Early detection allows for timely interventions, such as lifestyle modifications or pharmacological treatments, which can prevent or delay the onset of full-blown MetS and its associated complications. This proactive approach could lead to substantial improvements in public health outcomes and quality of life for at-risk individuals.

Moreover, our approach can be adapted for use in various populations, enhancing its generalisability and utility in diverse clinical settings. The flexibility of these models allows for recalibration based on population-specific characteristics, accounting for variations in genetic, environmental, and lifestyle factors across different regions or ethnic groups. This adaptability ensures that the models remain relevant and accurate when applied to populations that may differ from the original study cohort. The implementation of these predictive models in clinical practice could lead to more efficient allocation of healthcare resources. By identifying individuals at highest risk, healthcare providers can prioritize interventions and follow-up care, potentially reducing the overall burden on healthcare systems. Additionally, these models could serve as valuable tools for patient education and motivation, helping individuals understand their personal risk factors and encouraging proactive health management.

Our current study highlights the risk factors for predicting pre-MetS and MetS, but there are limitations and future research to consider. The focus on individuals with T2DM may limit the generalisability of our findings to non-diabetic populations. Validating predictive models and identified risk factors in a wider demographic, including non-diabetics and diverse ethnic and cultural groups, is essential [86, 90]. The cross-sectional nature of the study prevents establishing cause-and-effect relationships, highlighting the need for prospective longitudinal studies to explore these temporal associations and evaluate long-term predictive model for MetS [91–93]. Additionally, technology limitations, such as the need for high computational power, sophisticated software, and artificial intelligence expertise, pose challenges. Data entry errors and knowledge acquisition issues can lead to inaccuracies, while medical knowledge modelling and system performance need thorough validation [94]. Overcoming these limitations requires refining technology, ensuring cost-effectiveness, and establishing robust data management and system validation protocols.

Future research could focus on integrating these models into electronic health record systems, further streamlining the risk assessment process, and facilitating widespread adoption in various healthcare settings. This integration could support clinical decision-making and contribute to more personalised and effective patient care strategies. Additionally, further exploration to integrate genetic and lifestyle data into the predictive modelling framework will be innovative and worthwhile. Incorporating genetic information, including single nucleotide polymorphisms and gene expression data, may enhance the predictive accuracy and unravel the underlying biological mechanisms contributing to MetS development. The utilization of lifestyle factors, such as dietary patterns, physical activity levels, and sleep quality, could further refine the predictive models and provide a more comprehensive assessment of an individual’s cardiometabolic risk profile [95]. Another avenue for future investigation involves the development and validation of personalized intervention strategies based on the identified risk factors. For instance, individuals with elevated WHtR, VAI, and TyG index may benefit from tailored weight management programs, exercise regimens, and therapies targeting visceral adiposity and insulin resistance, whereas those with high LAP and dyslipidaemia may require personalized dietary modifications and lipid-lowering therapies [95–96]. Finally, as ML techniques continue to evolve, it would be valuable to explore the application of more advanced algorithms, such as deep learning and reinforcement learning, in predicting and managing MetS. These cutting-edge techniques have shown promise in various biomedical applications and may further enhance the predictive performance and provide insights into complex patterns within the data [97–99].

## Conclusion

Our study offers significant contributions to the field by integrating novel anthropometric indices to develop robust predictive models for predicting MetS. Our approach enhances predictive accuracy, provides a cost-effective screening tool for low-resource settings, and facilitates early intervention, and thus addresses a meaningful public health issue. We have demonstrated the potential of ML techniques and anthropometric indices in developing robust predictive models for pre-MetS and MetS. The ethical integration of these predictive models into clinical decision support systems or electronic health records could streamline the identification of high-risk individuals, enabling more efficient allocation of resources and targeted interventions. The identified risk factors, including LAP, WTI, CR, AVI, VAI, VLDLC and TyG-WHtR, provide valuable insights into the underlying visceral and metabolic dysregulation associated with MetS progression. Tackling these metabolic disturbances early on could empower healthcare professionals to mitigate or delay the onset of MetS, thereby reducing the impact of related cardiometabolic complications like cardiovascular disease, chronic kidney disease, and T2DM.

## Electronic supplementary material

Below is the link to the electronic supplementary material.

Supplementary Material 1

## Data Availability

The data and materials used in the study can be obtained by contacting the corresponding author with a reasonable request.
