# Supplementary material for: Predictive modelling of metabolic syndrome in Ghanaian diabetic patients: an ensemble machine learning approach
Source: J Diabetes Metab Disord. 2024 Aug 28;23(2):2233–49. doi: 10.1007/s40200-024-01491-7 (PMC11599523; doi:10.1007/s40200-024-01491-7)
Supplement: Supplementary file 1 — Supplementary Material 1 [file 40200_2024_1491_MOESM1_ESM.docx]

**Table 1: Characteristics of all ENRH, BDH, EGH and KATH cohort studies**

| Study characteristics | ENRH | BDH | EGH | KATH |
| --- | --- | --- | --- | --- |
| Study design | Cross-sectional study | Cross-sectional | Hospital-based cross-sectional study | Cross-sectional |
| Target population | Type 2 diabetes mellitus | Type 2 diabetes mellitus | Type 2 diabetes mellitus | Type 2 diabetes mellitus |
| Study location | Effia Nkwanta Regional hospital, Diabetic clinic | Begoro district hospital, Diabetic clinic | Ejisu Government Hospital, Out-Patient Department | Komfo Anokye Teaching Hospital, Diabetic clinic |
| Inclusion criteria | Age: ≥35 years, T2DM patient without pregnancy/lactating, drugs/alcohol abuse and chronic disease | Age >30 years, T2DM individuals with complete data on socio-demographical, lifestyle characteristics without chronic disease | Age: > 30 years, T2DM patient without pregnancy and chronic conditions | Age: 35 years, T2DM patient without pregnancy and chronic disease |
| Recruitment / sampling strategies | Face-to-face recruitment in diabetic clinic/non-probability sampling techniques | Face-to-face recruitment in diabetic clinic and out-patient department/ non-probability convenience sampling | Face-to-face recruitment, out-department department, convenience sampling | Face-to-face recruitment, Diabetic clinic, convenience sampling |
| Sample size | 196 | 387 | 242 | 282 |

T2DM: Type 2 diabetes mellitus

**Table 2: List of ENRH, BDH, EGH and KATH variables related to our study and selected for harmonisation**

| **Primary variables considered** | | **ENRH** | | **BDH** | | **EGH** | | **KATH** | | **Variable matching** | | **Harmonisation success** | |
| --- | --- | --- | --- | --- | --- | --- | --- | --- | --- | --- | --- | --- | --- |
| 1. Age | | Construct: Age at recruitment | | Construct: Age at recruitment | | Construct: Age at recruitment | | Construct: Age at recruitment | | Complete matching of construct | | Successfully harmonised | |
| 2. sex | | Data type: continuous, missing: . (period) | | Data type: continuous, missing: . (period) | | Data type: continuous, missing: . (period) | | Data type: continuous, missing: . (period) | | Complete matching of data response and coding except for missing value | | Data pooled into one variable (appended) | |
|  | | 1. Male | | 1. Male | | 1. Male | | 1. Male | |  | |  | |
|  | | 2.Female | | 2.Female | | 2.Female | | 2.Female | |  | |  | |
|  | | 3. . Missing | | 3. . Missing | | 3. . Missing | | 3. . Missing | |  | |  | |
| 3. Educational status | | Construct: EDUC LEVEL Data type: categorical | | Construct: Education Data type: categorical | | Construct: Education Data type: categorical | | Construct: Educational level Data type: categorical | | Partial matching of construct. Partial matching of response and coding | | Data harmonisation was not successful and could not be pooled into one variable | |
|  | | 1.Illiterate | | 1. Primary school | | 1. Primary school | | 1. Illiterate | |  | |  | |
|  | | 2. JSS | | 2. High school | | 2. Junior secondary school | | 2. Basic | |  | |  | |
|  | | 3.Middle school | | 3. Tertiary | | 3. Senior secondary school | | 3. Secondary | |  | |  | |
|  | | 4. Secondary school | |  | | 4. Tertiary | | 4. Tertiary | |  | |  | |
|  | | 5. Tertiary | |  | |  | |  | |  | |  | |
| 4. Duration of disease | | Construct: DOD/YRS. Data type: continuous Missing: . | | Construct: Duration of diabetes Data type: continuous Missing: . | | Construct: Duration of disease Data type: categorical Missing: . | | Construct: Duration of disease Data type: categorical Missing: . ( | | Partial matching of construct. Partial matching of response and coding, except missing value coding | | Data harmonisation was not successful and could not be pooled into one variable | |
|  | |  | |  | | < 5 | | < 0 | |  | |  | |
|  | |  | |  | | 5 10 | | 2 3 | |  | |  | |
|  | |  | |  | | > 10 | | 4 5 | |  | |  | |
|  | |  | |  | |  | | 5 10 | |  | |  | |
|  | |  | |  | |  | | 10 15 | |  | |  | |
|  | |  | |  | |  | | >15 | |  | |  | |
| 5. Height | | Construct: Height (cm) Data type: continuous Missing: . | | Construct: Height (m). Data type: continuous Missing: . | | Construct: height (cm) Data type: continuous Missing: . | | Construct: height (cm) Data type: continuous Missing: . | | Complete matching of construct Complete matching of data response and coding except for missing value | | Successfully harmonised Data pooled into one variable (appended) | |
| 6. Sex | | Construct: Gender status Data type: categorical | | Construct: Sex status Data type: categorical | | Construct: Sex status Data type: categorical | | Construct: Sex status Data type: categorical | | Complete matching of construct Complete matching of data response and coding except for missing value | | Successfully harmonised Data pooled into one variable (appended) | |
|  | | 1. Male | | 1. Male | | 1. Male | | 1. Male | |  | |  | |
|  | | 2.Female | | 2.Female | | 2.Female | | 2.Female | |  | |  | |
|  | | 3. . Missing | | 3. . Missing | | 3. . Missing | | 3. . Missing | |  | |  | |
| 7. Weight | | Construct: Weight (kg) Data type: continuous Missing: . | | Construct: Weight (kg) Data type: continuous Missing: . | | Construct: weight Data type: continuous Missing: . | | Construct: Weight (kg) Data type: continuous Missing: . | | Complete matching of construct Complete matching of data response and coding except for missing value | | Successfully harmonised Data pooled into one variable (appended) | |
| 8. Waist circumference (WC) | | Construct: WC (cm) Data type: continuous Missing: . | | Construct: Waist circumference Data type: continuous Missing: . | | Construct: WC Data type: continuous Missing: . | | Construct: Waist circumference (cm) Data type: continuous Missing: . | | Complete matching of construct Complete matching of data and coding except for missing value | | Successfully harmonised Data pooled into one variable (appended) | |
| 9. Hip circumference (HC) | | Construct: HC (cm) Data type: continuous Missing: . | | Construct: Hip circumference Data type: continuous Missing: . | | Construct: HC Data type: continuous Missing: . | | Construct: Hip circumference (cm) Data type: continuous Missing: . | | Complete matching of construct Complete matching of data and coding except for missing value | | Successfully harmonised Data pooled into one variable (appended) | |
| 10. Occupational status | | Construct: OCCUP Data type: categorical | | Construct: Occupation Data type: categorical | | Construct: Occupation status Data type: categorical | | Construct: Occupational status Data type: categorical | | Partial matching of construct. Partial matching of response and coding | | Data harmonisation was not successful and could not be pooled into one variable | |
|  | | 1. Unemployed | | 1. None | | 1. Government employee | | 1. Informal | |  | |  | |
|  | | 2. Formal | | 2. Self-employed | | 2. Private employee | | 2. Formal | |  | |  | |
|  | | 3. Self-employed | | 3. Government employed | | 3. Self -employed | | 3. Retired | |  | |  | |
|  | | 4.Retired | | 4. Retired | | 4. Unemployed | |  | |  | |  | |
| 11. Systolic blood pressure (SBP) | | Construct: SBP (mmHg) Data type: continuous Missing: . | | Construct: SBP (mmHg) Data type: continuous Missing: . | | Construct: SBP Data type: continuous Missing: . | | Construct: SBP Data type: continuous Missing: . | | Complete matching of construct Complete matching of data and coding except for missing value | | Successfully harmonised Data pooled into one variable (appended) | |
| 12. Diastolic blood pressure (DBP) | | Construct: DBP (mmHg) Data type: continuous Missing: . | | Construct: DBP (mmHg) Data type: continuous Missing: . | | Construct: DBP Data type: continuous Missing: . | | Construct: DBP Data type: continuous Missing: . | | Complete matching of construct Complete matching of data and coding except for missing value | | Successfully harmonised Data pooled into one variable (appended) | |
| 13. Fasting blood sugar (FBS) | | Construct: FBS (mmol/L) Data type: continuous Missing: . | | Construct: FBS (g/dL) Data type: continuous Missing: . | | Construct: FBS (mmol/L) Data type: continuous Missing: . | | Construct: FBS. Data type: continuous Missing: . | | Complete matching of construct Complete matching of data and coding except for missing value | | Successfully harmonised Data pooled into one variable (appended) | |
| 14. Triglyceride (TG) | | Construct: TG (mmol/L) Data type: continuous Missing: . | | Construct: TG (g/dL) Data type: continuous Missing: . | | Construct: TG (mmol/L) Data type: continuous Missing: . | | Construct: TG. Data type: continuous Missing: . | | Complete matching of construct Complete matching of data and coding except for missing value | | Successfully harmonised Data pooled into one variable (appended) | |
| 15. High-density lipoprotein cholesterol (HDL-C) | | Construct: HDL-C (mmol/L) Data type: continuous Missing: . | | Construct: HDL-C (g/dL) Data type: continuous Missing: . | | Construct: HDL-C (mmol/L) Data type: continuous Missing: . | | Construct: HDL-C. Data type: continuous Missing: . | | Complete matching of construct Complete matching of data and coding except for missing value | | Successfully harmonised Data pooled into one variable (appended) | |
| 16. Total Cholesterol (TC) | | Construct: TC (mmol/L) Data type: continuous Missing: . | | Construct: TC (g/dL) Data type: continuous Missing: . | | Construct: Total cholesterol (mmol/L) Data type: continuous Missing: . | | Construct: TC. Data type: continuous Missing: . | | Complete matching of construct Complete matching of data and coding except for missing value | | Successfully harmonised Data pooled into one variable (appended) | |

**Table 3: Descriptive characteristics of all ENRH, BDH, EGH, KATH and combined cohort**

| Variables | ENRH (n=196) | BDH (n=387) | EGH (n=242) | KATH (n=282) | Combined |
| --- | --- | --- | --- | --- | --- |
|  | Mean (95% CI) | Mean (95% CI) | Mean (95% CI) | Mean (95% CI) | Mean (95% CI) |
| Age (years) | 50.4 (49.5 - 51.4) | 56.4 (53.1 - 58.8) | 57.5 (56.2 - 58.8) | 50.7 (49.9 - 51.6) | 53.2(52.5 – 53.8) |
| Height (m) | 1.65 (1.64 - 1.66) | 1.61 (1.59 - 1.62) | 1.60 (1.59 - 1.61) | 1.62 (1.61 - 1.63) | 1.62(1.57 – 1.68) |
| Weight (kg) | 73.2 (71.3 - 75.2) | 67.1 (64.9 - 69.3) | 71.3 (69.6 - 73.1) | 72.4 (71.0 - 73.9) | 71.6(70.7 – 72.5) |
| Waist circumference (cm) | 95.3 (93.4 - 97.1) | 91.9 (89.9 - 93.9) | 92.8 (91.1 - 94.4) | 93.4 (92.2 - 94.7) | 93.5(92.7 – 94.3) |
| Hip circumference (cm) | 106(104.4 - 107.8) | 100.1 (98.2 - 101.9) | 99.4 (97.7 - 101.1) | 103.8 (102.6 - 105.1) | 102.6(101.8 – 103.5) |
| Waist-height ratio | 0.59 (0.58 - 0.60) | 0.57 (0.56 - 0.59) | 0.56 (0.55 - 0.57) | 0.57 (0.56 - 0.58) | 0.57(0.56 – 0.58) |
| Waist-hip ratio | 0.89 (0.88 - 0.90) | 0.92 (0.91 - 0.93) | 0.93 (0.92 - 0.94) | 0.91 (0.89 - 0.93) | 0.92(0.91 – 0.94) |
| Body mass index (kg) | 28.5 (27.7 - 29.3) | 26.2 (25.3 - 27.1) | 26.2 (25.6 - 26.9) | 27.5 (26.9 - 28.0) | 27.2(26.8 – 28.3) |
| Body adiposity index | 34.4 (33.3 - 35.2) | 31.5 (30.4 - 32.6) | 29.0 (28.1 - 29.9) | 32.2 (31.6 - 33.0) | 31.7(31.2 – 32.2) |
| Conicity index | 1.29 (1.28 - 1.31) | 1.31 (1.29 - 1.32) | 1.30 (1.28 - 1.32) | 1.29 (1.28 - 1.30) | 1.29(1.28 – 1.31) |
| Abdominal volume index | 18.6 (17.9 - 19.3) | 17.2 (16.5 - 17.9) | 17.6 (17.0 - 18.2) | 18.1 (17.6 - 18.5) | 17.9(17.6 – 18.2) |
| Visceral adiposity index | 2.11 (1.95 - 2.28) | 2.48 (1.97 - 2.99) | 1.44 (1.27 - 1.62) | 1.55 (1.43 - 1.67) | 1.76(1.66 – 1.86) |
| Waist triglyceride index | 7.25 (7.20 - 7.30) | 7.24 (7.16 - 7.32) | 6.88 (6.82 - 6.94) | 6.93 (6.88 - 6.99) | 7.02(6.99 – 7.06) |
| Lipid accumulation product | 55.0 (50.4 - 59.6) | 51.4 (44.9 - 57.9) | 51.8 (48.4 - 55.2) | 57.3 (44.0 - 60.6) | 44.1(42.0 – 46.3) |
| Triglyceride-glucose index | 7.78 (7.69 7.86) | 7.89 (7.79 - 8.00) | 7.36 (7.36 - 7.45) | 7.41 (7.34 - 7.47) | 7.54(7.49 – 7.58) |
| TyG-BMI | 221.7 (215.0 - 228.4) | 207.3 (199.4 - 215.2) | 192.5 (187.6 - 197.6) | 203.7 (199.1 208.2) | 205.1(202.2 – 213.1) |
| TyG-WC | 741.7 (724.5 - 758.8) | 726.2 (707.7 - 744.6) | 682.1 (668.0 - 696.2) | 692.8 (681.4 - 704.3) | 704.9(697.6 – 712.3) |
| TyG-WHR | 6.96 (6.86 - 7.07) | 7.26 (7.13 - 7.39) | 6.88 (6.78 - 6.98) | 6.77 (6.62 - 6.92) | 6.91(6.83 – 6.97) |
| TyG-WHtR | 4.63 (4.52 – 4.74) | 4.54 (4.42 – 4.66) | 4.14 (4.05 – 4.23) | 4.27 (4.20 – 4.34) | 4.34(4.29 – 4.40) |
| Systolic blood pressure (mmHg) | 137.8 (135.2 - 140.5) | 133.3 (129.7 - 136.9) | 139.3 (136.2 - 142.4) | 119.0 (117.7 - 120.2) | 130.2(128.8 – 131.5) |
| Diastolic blood pressure (mmHg) | 83.6 (82.1 - 85.1) | 81.1 (78.8 - 83.4) | 81.5 (79.8 - 83.3) | 72.7 (71.8 - 73.7) | 78.4(77.6 – 79.0) |
| Fasting blood sugar (mmol/L) | 9.89 (9.18 - 10.6) | 10.5 (9.76 - 11.3) | 9.27 (8.69 - 9.85) | 8.76 (8.43 - 9.09) | 9.36(9.09 – 9.64) |
| Triglyceride (mmol/L) | 1.74 (1.65 – 1.82) | 1.86(1.69 – 2.03) | 1.28 (1.20 – 1.36) | 1.41 (1.33 – 1.49) | 1.51(1.46 – 1.56) |
| Total cholesterol (mmol/L) | 5.20 (5.02 - 5.39) | 6.90 (6.61 - 7.18) | 4.75 (4.59 - 4.92) | 5.16 (5.02 - 5.30) | 5.29(5.19 – 5.38) |
| High-density lipoprotein (mmol/L) | 1.18 (1.14 - 1.23) | 1.24 (1.17 - 1.31) | 1.34 (1.30 - 1.39) | 1.36 (1.32 - 141) | 1.30(1.28 – 1.33) |
| Low-density lipoprotein (mmol/L) | 3.67 (3.51 - 3.83) | 4.85 (4.58 - 5.12) | 2.81 (2.66 - 2.96) | 3.36 (3.23 - 3.49) | 3.47(3.38 – 3.56) |
| Coronary risk | 4.63 (4.42 - 4.84) | 6.05 (5.57 - 6.54) | 3.74 (3.54 - 3.94) | 4.28 (4.05 - 4.50) | 4.44(4.30 -4.57) |
| Vey low-density lipoprotein | 0.79 (0.75 - 0.82) | 0.85 (0.77 - 0.92) | 0.58 (0.55 - 0.62) | 0.64 (0.60 - 0.68) | 0.68(0.66 – 0.71) |
